# Dr. Venables' Clinical Report on Dropsies; with an Appendix on the Theory and Treatment of Organic Disease in General

**Published:** 1825-07-01

**Authors:** 


*Clinical Report on Dropsies; with Observations explanatory of their Pathology and Therapeutics: with an Appendix on the Theory and Treatment of Organic Disease in general.* By Robert Venables, Bachelor in Medicine, &c. &c. Octavo, pp. 240, Underwoods, London, 1824.

Dr. Venables, we apprehend, is writing rather too fast and too soon. "*Festina lente*," is no bad precept in authorship; and, doubtless, the rough salutation which Dr. Venables has met with among our critical brethren may, ere this, have inclined him to think so. Not that we coincide in the severe strictures which have been passed on the work before us. We think it is, like most productions in this world, a mixture of good and evil—of truth and error, from which something useful may be gleaned, and of which a good deal must be rejected. Our author appears to pin his faith too much, for an original observer, on the tenets of one or two modern writers—nor is he very happy in conveying his conceptions to the understandings of his readers. Thus, at page six of his preface he observes:—" The regarding fever as *symptomatic* rather than the *cause* of local affections, has tended much to obscure both their nature and theory, and to enervate our practice." Surely this sentence would lead us to conclude that Dr. V. did *not* regard fever as the *effect*, but rather the *cause* of the topical affection. And yet a few lines farther on we have the following confused enunciation.

" I have long regarded febrile and inflammatory action as the same in *essence*,* and differing merely in the extent of parts occupied by the morbid action. Let fever, then, and the diseases in which febrile action prevails, or which are accompanied with these morbid manifestations, be treated as other inflammations of *a similar description* would be, and I am convinced, that diseases which are now regarded as obstinate and incurable, will become infinitely more tractable, and, in many instances, yield to medical treatment." vii.

Here, then, fever and inflammation are identified, instead of standing in the relation of cause and effect—and fevers are to be treated as "*other* inflammations of a *similar kind*." What can our author possibly mean by comparing or coupling fevers with *other* inflammations of a *similar kind*? But, indepen-

" * Vide Wilson Philip's Essay on Fever."

dently of the discrepancy and obscurity of these passages, we do maintain that the tendency of the doctrine is most dangerous. The general treatment of fever and inflammation never can be amalgamated without great detriment to the patient. *Fever* will be cured by various and *opposite* methods of treatment—and, perhaps, in a very overwhelming majority of cases it would terminate safely without any medicine at all. Not so inflammation of an internal organ. There is not, indeed, according to our apprehension, a more powerful argument against the identity of fever and inflammation than this difference in treatment—a difference which is more and more conspicuous in proportion to our clinical experience.

Before entering on his clinical report, Dr. V. makes some observations on the sense which he attaches to the word *pathology.* He distinguishes it, and very properly, from morbid anatomy, with which it is sometimes confounded. Pathology, as the name imports, is *a discourse on disease.* In its more general signification, it not only includes the *morbid action* going on in disease, but their causes, and even their symptoms: morbid anatomy, on the other hand, means only the *deviations from healthy structure,* caused by the morbid actions, and remaining when these actions and life itself have ceased.

Dr. V.'s clinical report consists of the detail of twenty-one cases, with remarks on individual cases; to which are appended inferences and general observations on the disease in question. The cases were, in general, of the same kind as those detailed by Dr. Crampton, and treated on the same antiphlogistic principles. We shall give a single case, as a specimen, and then pass on to the author's general deductions.

"*Case* 10. Frances Avery, aged 47, a thin, poor, spare-looking woman.—Dispensary.

"September 10, 1823. Complains of a most severe and distressing dyspnœa; 'can scarcely get her breath;' any exercise, such as merely walking across the room, creates such a degree of dyspnœa, that she gasps and catches for air; violent fits of coughing follow, which last for ten or fifteen minutes; orthopnœa; the face flushes and becomes purple, the lips assume a livid hue, and the whole countenance indicates the greatest distress. The skin feels hot, dry, and rough to the touch; the pulse quick, irregular, hard, and slow; only 50 beats in the minute. The bowels constipated; the urine scanty and high-coloured, and on settling deposits a sediment; does not coagulate on the application of heat; tongue foul, and covered with a yellow fur; the abdomen swoln, and, on percussion, fluctuation distinctly perceived. Anasarca of the ancles, with pitting on pressure. *Mitt. sanguis ad* ℥x. *Ht. pil. col. comp. no.* vj.—*st.* j. *sing. horis, ad alvum promovend.* ℞ *Pulv. digitalis* gr. xij. *pulv. ant.* gr. xx. *ipecacuanhæ* gr. x. *confecti. aromat. q. s. ut ft. pil.* xij.—*st.* j. *bis in dic.*

"12. The symptoms detailed above, nearly as at the time of that report. Complains of restless nights, palpitations at the heart, and frequent attacks of night-mare, with startings from sleep. Has taken her medicine with very little relief, or even perceptible effect. The aperient pills have regulated the bowels. The blood drawn exhibited, on standing, signs of inflammatory action. *Perstat, et admov. hirud. no.* x. *sterno, et posteà vesicatorium.*

"17. Symptoms somewhat relieved; the dyspnœa and coughing not so violent; the restlessness and palpitations continue, though she thinks they are not so severe or violent as before. *Perstat; et st. ext. hyoscyami* gr. iij *horâ somni, et repet. cras vespere.*

"19. All the symptoms abated. The evacuations, however, somewhat dark-coloured. ℞ *Calomel.* gr. vj. *pulv. digitalis* gr. x. *ext. hyoscyami* gr. viij. M. *ft. pil.* xij.—*st.* j. *nocte maneque.*

"24. Some increase of the dyspnœa; the symptoms generally, however, improved; the cough is not so readily excited; the orthopnœa relieved, as she can now lie down in nearly a recumbent posture. *Admov. vesicatorium sterno, et perstat ut antè.*

"26. All the symptoms rapidly disappearing; the abdomen decreases in size; the urine plentiful; the bowels free; the fever and pectoral symptoms very much abated. *Perstat.*

"October 3. All the symptoms completely relieved. She feels weak, and sometimes the urine diminishes in quantity. ℞ *Infusi cascarillæ* ℥viij. *infusi digitalis* ℥ij. M.—*st. cyath.* j. *ter in die.*

"20. Has not attended since till this day; complains of a return of the symptoms; the urine diminished; pyrexia, with hard pulse. *Mitt. sanguis ad* ℥x.* ℞ *Cremor. tart.* ʒij. *jalap.* ℈ij. M. *ft. pulvis, cujus sumat* gr. xij. *ter in die.*

"27. The powders purge very much; the urine not quite so plentiful. ℞ *Cremor. tart.* ℥ss. *jalap.* ʒjss. *gum kino* ʒss. *theriacæ q. s. ut ft. elect.*—*st. coch.* j. *min. bis terve in die.*

"She persevered in the above medicine till the 12th Nov. when, through an imprudent exposure to cold while washing, she was again attacked with acute febrile symptoms, which threatened a return of all her former distress. Eight ounces of blood were now drawn off, and the electuary directed to be repeated.

"Nov. 17. The bleeding immediately checked the fever, and she now feels perfectly well, with the exception of weakness, from the active treatment to which she has been subjected. She was now directed to take the tartarized iron, as a tonic, and was discharged, cured, on the 24th of the month.

"*Observations.*—This was as severe a case of hydrothorax as I have witnessed. The appearance of this woman was not what would be considered as warranting bleeding. The pulse was unusually slow, but, after bleeding, it rose, and acquired a much greater frequency.

"* The blood drawn from this patient did not buff in any instance."

This often happens; when the quantity of blood is such as to oppress the heart, or that there is such pulmonary obstruction as to impede the circulation through these organs, the motion of this fluid becomes preternaturally slow. Venesection, by relieving the actual disease, and lessening the influence of its causes, removes the obstruction, and the blood acquires a greater degree of velocity. I am much inclined to believe that the lungs, in this case, were in that morbid condition which Laennec has termed 'Pulmonary Apoplexy.'* Although hæmoptoe is a general attendant on this condition of the lungs, yet I question whether it be an absolutely necessary consequence. However, I questioned this woman upon the subject of spitting of blood, but she answered in that hesitating and equivocal manner which led me to doubt the accuracy of her assertions. Some people seem to imagine, that to admit of being afflicted with disease is a sure mode of becoming affected; perhaps she thought the converse of this equally good reasoning, and that to deny the presence of symptoms was a sure way of diminishing their violence.

"It will be observed, that a frequent repetition of the blood-letting and blistering was absolutely necessary, to establish any thing like permanent relief.† Surely it could not be to the want of diuretics, nor to a diminished secretion of urine, that the aggravation of the symptoms, which occasionally took place in this instance, should be attributed. The increase of the symptoms was usually attended with pyrexia; this proved a stimulus to the heart, and excited inordinate action.‡ The impermeability of the pulmonary tissue to the blood, propelled with an unusual velocity, no doubt, contributed much to the dyspnœa, cough, and other kinds of pectoral distress experienced in this case." 57.

From the detail of the 21 cases of dropsy, our author considers himself fully justified in deducing the following inferences.

"1. Dropsy is more frequently an active disease.

"2. When active, it is generally complicated with a pyrexial or inflammatory state of the system.

"3. When dropsy depends upon inflammatory affections of the viscera, the operation of these affections is most frequently indirect; namely, through the fever which commonly accompanies such diseases.

"4. Dropsy may arise as the termination of acute and subacute inflammation in the serous membranes, or in the cellular structures.

"5. Dropsy, in a very severe degree, may exist, independently of any organic disease.

"6. When the cellular structures, or serous membranes, &c. are

"* See Laennec, translated by Dr. Forbes: and also Dr. Forbes's own work on the Stethoscope and Percussion, &c."

"† I have since seen this woman repeatedly at church, and she seems to enjoy very good health."

"‡ At least, in a comparative sense."

weak, either naturally from mal-conformation, from disease, or mechanical violence, the excitement of the economy, during the earlier periods of pregnancy,* may lead to a dropsy of the weakened parts, which, if neglected, will, under unfavourable circumstances, become alarming and inveterate diseases.

"7. When dropsy exists, in combination with an excited state of the system, or with an inflammatory affection of any organ or texture, antiphlogistic measures, especially blood-letting and antimonials, form the most rational and effectual means of cure.

"8. A reliance on diuretics in active dropsies serves merely to debilitate the system, without curing the disease, and may even lead to diabetes.

"9. A coagulable state of the urine in dropsy generally indicates the necesssity of blood-letting; but the converse of this proposition does not hold good, and the non-coagulability of the urine does not necessarily prohibit venesection." 124.

We do not see any thing very objectionable in the foregoing inferences. They are confirmed by the cases themselves, and, indeed, by the general observation of attentive practitioners. After the able works, however, which have been lately published on the subject of inflammatory dropsy, we think there is not sufficient of novelty in Dr. Venables' views to require a formal volume for their enunciation. A concise essay in a periodical journal would have answered every purpose. We are free to confess, however, that a perusal of the cases and remarks on them, has not brought to our view any good grounds for those severe criticisms which have been so plentifully bestowed on this clinical report.

*Theory and Treatment of Organic Disease.*

What is organic disease? Most people readily conceive what is meant by organic disease—namely, *a cognizable deviation from the natural or healthy structure of a part*. Functional disorder, on the other hand, is a deviation from natural or healthy office in a part, unaccompanied by any *perceptible* change in the structure. There can be little doubt, however, that these two states are divided by imaginary rather than real boundaries. We can hardly suppose an organ to be out of or-

---

"* Of course, excitement of any kind will, under the same circumstances, lead to similar results. But pregnancy being a natural process, the excitement generally attending it would be suffered to pass unobserved and unheeded; whereas a much inferior degree of excitement, arising from an unnatural or unusual cause, would obtain serious attention. I therefore thought it necessary to notice specifically the possible consequences from pregnancy."

der, in function, say the stomach, without some change in its nervous or vascular system—although such change is not cognizable after death. Functional disorder then, and organic disease are rather two *stages* than two distinct *states* or conditions. Still, admitting this connexion, and also the well-known fact, that functional disorder often terminates in organic disease, the distinction is proper and useful in practice. As long as there is no material change in structure, beyond that of common inflammation, we have every reason to expect a cure—but when material change of organization has once obtained, the chance of cure is then indeed but small. We have no objection to the following arrangement of Dr. V.

"Then the animal mechanism may be considered susceptible of three degrees of change: the first consisting in such a change as has been generally named irritation, and which Mr. Abernethy has termed disorder; the second degree comprehends those changes which are the result of the less severe inflammatory affections, and which, perhaps, may be termed disease; the third change includes those alterations which render the structure itself worse than useless, for it becomes a perpetual source of irritation, and, generally speaking, ultimately proves fatal." 147.

The first change in the animal mechanism (functional disorder) is either in itself so minute, or takes place in the ultimate particles placed beyond the sphere of our vision, that it is in no way cognizable by the senses. It is only to be inferred by the phenomena or symptoms which result from it. This disorder is generally referred to the nervous system. But, considering the connexion and necessary dependence of the nervous and vascular systems, we can hardly suppose that any one is long deranged without corresponding derangement in the other. It is well known that the late Dr. Parry, of Bath, was inclined to attribute the first movements of disorder to the vascular rather than to the nervous system, and Dr. V. himself appears disposed to adopt the same views. But this has always been considered as one of the weakest points in Dr. Parry's doctrines. For although changes in the vascular apparatus almost invariably and inevitably affect the sentient structure, still if we analyze minutely the phenomena of healthy as well as disordered actions, we shall come to the conclusion, that impressions (whether from external agents or internal emotions) are first made on the nerves, and through them on the vascular apparatus.

Under the head of inflammation, (as the great source of organic disease) our author does little else than copy, page after page, from Dr. Philip's work on indigestion. One would indeed suppose that Dr. Venables had kept Dr. Philip's work under his pillow, to study it by night as well as read it by day. Considering how many editions of it have issued from the press, we really think it was unnecessary to republish it on the present occasion. It would be still less necessary for us, in this place, to select the "*disjecta membra*" of Abernethy and Philip for specimens of Dr. Venables. Our author has completely debarred us from making any extracts, or offering any analysis of his appendix on organic diseases.

